# Paraoxonase Role in Human Neurodegenerative Diseases

**DOI:** 10.3390/antiox10010011

**Published:** 2020-12-24

**Authors:** Cadiele Oliana Reichert, Debora Levy, Sergio P. Bydlowski

**Affiliations:** 1 Lipids, Oxidation, and Cell Biology Group, Laboratory of Immunology (LIM19), Heart Institute (InCor), Hospital das Clínicas HCFMUSP, Faculdade de Medicina, Universidade de São Paulo, São Paulo 05403-900, Brazil; cadiele@usp.br (C.O.R.); d.levy@hc.fm.usp.br (D.L.); 2 Instituto Nacional de Ciencia e Tecnologia em Medicina Regenerativa (INCT-Regenera), CNPq, Rio de Janeiro 21941-902, Brazil

**Keywords:** paraoxonases, oxidative stress, multiple sclerosis, amyotrophic lateral sclerosis, Alzheimer’s disease, Parkinson’s disease

## Abstract

The human body has biological redox systems capable of preventing or mitigating the damage caused by increased oxidative stress throughout life. One of them are the paraoxonase (PON) enzymes. The PONs genetic cluster is made up of three members (PON1, PON2, PON3) that share a structural homology, located adjacent to chromosome seven. The most studied enzyme is PON1, which is associated with high density lipoprotein (HDL), having paraoxonase, arylesterase and lactonase activities. Due to these characteristics, the enzyme PON1 has been associated with the development of neurodegenerative diseases. Here we update the knowledge about the association of PON enzymes and their polymorphisms and the development of multiple sclerosis (MS), amyotrophic lateral sclerosis (ALS), Alzheimer’s disease (AD) and Parkinson’s disease (PD).

## 1. Introduction

Over the years, biotechnological changes and advances have guaranteed the population a significant increase in life expectancy that does not necessarily involve an increase in quality of life and/or having a healthy old age. The human body is a complex organism that maintains a balance of essential biochemical–physiological functions. When this balance is broken, the human body acts to restore homeostasis. However, in certain situations this is not possible, and as a biological characteristic damaged tissue is observed to be accompanied by loss of function and cell death. Such events can occur in any part of the human body: Cutaneous, skeletal, muscular, cardiovascular, respiratory, digestive, urinary, genital and nervous systems. When irreparable damage is observed in the nervous system, the neurodegeneration process is established. The signs and symptoms are noticeable in the short to long term, depending on the location in the central nervous system (CNS) where the damage has started. Aging is considered a risk factor for the onset of the degenerative process. For instance, currently around 50 million people live with dementia, and it is expected that by the year 2050 this number will triple (approximately 152 million people) [1,2].

**Figure 1:**
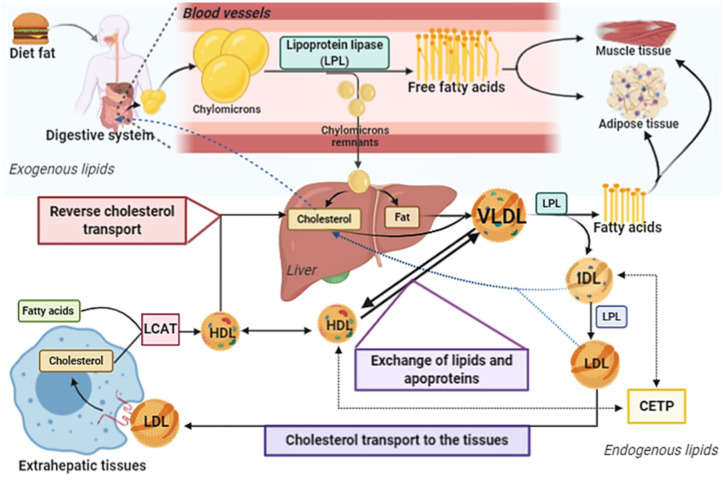
First, dietary fat is emulsified by bile salts, being degraded by lipases. Fatty acids are absorbed through the intestinal mucosa, being converted into triacylglycerol. Then there is the joint incorporation of cholesterol, triglycerides and apolipoproteins (Apo-CII and Apo-B48), forming the chylomicrons. Dietary fat is transported in the blood and lymph vessels by the chylomicrons. Apo-CII on the surface of chylomicron activates the enzyme lipoprotein lipase (LPL), which releases fatty acids and glycerol to tissues, mainly muscle and adipose tissues. In tissues, fatty acids can be esterified and stored or used to generate energy in cellular function. This process results in the formation of chylomicron remnants (Apo-B48 and Apo-E in surface), that have cholesterol and low triglyceride content. They are transported to the liver, where receptors bind to the Apo-E, leading to endocytosis and subsequent degradation in lysosomes, with the release of cholesterol and fatty acids. In the liver, very-low density lipoprotein (VLDL) is formed. It is rich in triacylglycerols (about 55%) and contains on its surface the following Apos: B100, E and C I, II, III. The Apo-C and -E in VLDL come from plasma high-density lipoproteins (HDL). In addition, during the lipolysis process, Apo-C, cholesterol and phospholipids are transferred from VLDL to HDL. LPL hydrolyzes VLDL in capillaries releasing fatty acids to tissues. The hydrolysis process gives rise to the IDL (or remaining VLDL), with Apo-E and Apo-B100 on its surface. IDLs are endocytosed in the liver, after binding to the low-density lipoprotein (LDL) (Apo-B100) or LDL receptor related protein (LRP) (binding to the Apo-E). IDL is also converted to LDL (about 50% cholesterol) by the action of liver LPL, which contains only one Apo-B100 molecule on its surface. This mechanism is not yet fully described. However, it is believed that there is an exchange of apolipoproteins and lipid content between HDL and IDL, by cholesterol ester transfer protein (CETP), together with phospholipid and triacylglycerol hydrolysis by hepatic LPL for the formation of LDL. LDL transports cholesterol to the extra-hepatic tissues by the binding of Apo-B100 to the LDL receptor or scavenger receptors followed by endocytosis. LDL receptor expression is downregulated by the content of intracellular cholesterol. Cholesterol is transported from tissues to the liver by HDL, which contains apolipoproteins A, C and E. In tissues, the ATP-binding cassette (ABC)-A1 and G1 proteins participate of the transport of cholesterol to HDL. Cholesterol in plasma is esterified by lecithin-cholesterol acyl transferase (LCAT), an enzyme activated by apolipoprotein-AI (Apo-AI). LCAT, in conjunction with lecithin (present in HDL), catalyzes the formation of cholesterol esters from fatty acids. As HDL captures cholesterol from tissues or lipoprotein hydrolysis, its diameter increases, from nascent HDL to discoid HDL. When the cholesterol ester content is taken, HDL migrates to the liver, binds to the Apo-AI receptor, and is released in hepatocytes. Then, cholesterol can originate VLDL or be excreted via the biliary or fecal pathways.

The etiology of the several neurodegenerative diseases is still unclear, being multifactorial [3,4]. First, there are different neurodegenerative diseases, since the CNS is composed of different cell populations, in different areas, with highly specialized and unique functions. However, some risk factors are common among these diseases, such as: Exposure to certain toxins; presence of certain polymorphisms; changes in cholesterol metabolism; decreased antioxidant activity and increased oxidative stress. All of these factors together favor the loss of function and death of nerve cells [5,6,7,8]. The transport of human cholesterol is complex and joint integration between lipoproteins, enzymes and apolipoproteins (Apo) is necessary, Figure 1. Free cholesterol can be easily oxidized by reactive oxygen species (ROS), giving rise to a group of compounds called oxysterols. Oxysterols participate in several pathophysiological processes such as drug resistance, stem cell differentiation, cell proliferation and death [9,10,11,12,13,14,15,16]. They are also inducers of neuroinflammation and have a role in neurodegenerative diseases [17,18].

Another factor associated with neurodegenerative diseases is the increase in oxidative stress in the CNS. Oxidative processes of cellular metabolism lead to the formation of reactive oxygen or nitrogen species (RNS), due to the partial reduction in molecular oxygen (O_2_) by both free electrons and radicals [19,20]. The primary ROS products generated after the partial reduction of O_2_ are singlet oxygen (1O_2_), O_2_^•−^ and H_2_O_2,_ while subsequent reactions generate hydroxyl radical (OH•) and hypochlorous acid (HOCl) [19,20]. ROS and free radicals induce progressive damage to macromolecules such as DNA, lipids, carbohydrates and proteins [21,22]. Increased ROS interfere with cell signaling, leading to several metabolic changes, including modification in the permeability and fluidity of phospholipid membranes. In addition, active and passive transport of compounds and substrates through membrane cell is also affected [21,22].

The human body has many enzymatic systems for the protection of genotoxic damage, such as cytochrome P450, and directly or indirectly through free radical scavenging, such as paraoxonase (PON) [21]. Paraoxonases protect HDL and LDL from oxidative stress by removing ROS produced by the metabolism [23]. Here, we present the main evidence described in humans linking paraoxonase enzymes to some of the most frequent neurodegenerative diseases, discussing possible mechanisms of action.

## 2. Paraoxonase Family

The paraoxonases family consists of three enzymes: Paraoxonase 1 (PON1), paraoxonase 2 (PON2) and paraoxonase 3 (PON3), all having antioxidant and hydrolase activities. Although PON enzymes are widely distributed throughout the human body, these enzymes are mainly synthesized in the liver. They are present in different tissues, and are mainly associated with cell membranes and some lipoproteins, although free enzyme was described in the blood.

**Figure 2:**
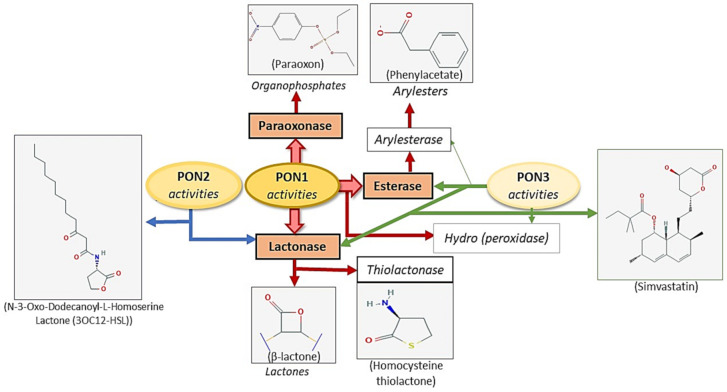
Paraoxonase family activities and substrates.

Historically, paraoxonase was named after its ability to hydrolyze paraoxon, a compound of the organophosphate insecticides class, to the metabolite p-nitrophenol [24]. In vivo, paraoxon, the most toxic form, is an oxidized product of biotransformation of parathion [24]. In fact, the PON family is able to metabolize other compounds such as: Plucuronide drugs, lactone compounds, arylesters, aromatic carboxylic acid and unsaturated aliphatic esters, cyclic carbonate, nerve gases and some carbamate insecticide classes. Figure 2. Furthermore, PON inactivates lipoxidation derivatives of low-density lipoprotein (LDL) [25,26,27].

PON1, PON2 and PON3 genes are located adjacent to each other on human chromosome seven, having about 80%–95% similarity; the proteins share 79–95% amino acid sequence [28]. All PON genes have nine exons, eight introns and TATA-less promoters [29]. Much of our understanding on paraoxonases comes from studies related to PON1.

### 2.1. Paraoxonase 1 (PON1)

Paraoxonase 1 is a calcium-dependent glycoprotein of 354 amino-acid, with a molecular weight of 43-47kDa. In humans, PON1 is encoded in the chromosome seven (7q21.3–22.1), synthesized mostly in the liver, and in small quantities in the small intestine and kidneys [30,31]. PON1 was first identified in mammals during the 1950s [32]. It has been found in others animals, although its activity is reduced [32,33,34,35].

**Figure 3:**
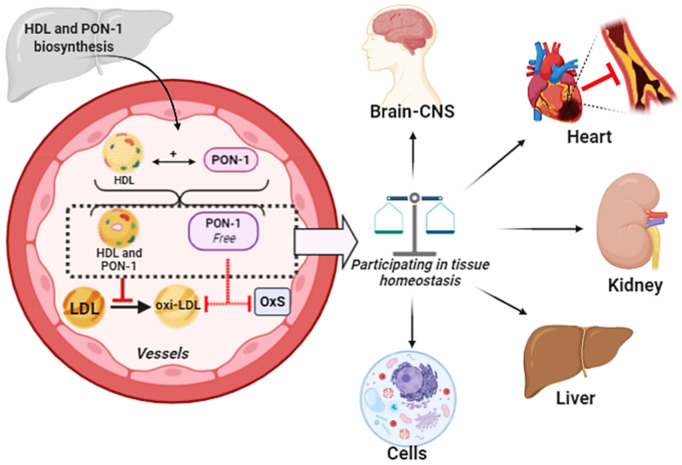
HDL biosynthesis occurs in the liver and in a small part of the intestine. Paraoxonase 1 (PON1) biosynthesis occurs only in the liver. After the formation of both HDL and PON1, they enter into the circulation. An association between HDL and PON1 can occur both in the hepatic circulation (to a lesser extent) and in the plasma. A small amount is free PON1, which do not adhere to any lipoprotein. An important function of PON1 is to prevent oxidation of both HDL and LDL, through the hydrolysis of reactive compounds. Oxidized LDL (ox-LDL) is pro-inflammatory and atherogenic. PON1 is carried to the tissues by HDL, where it performs its function as an antioxidant enzyme. In addition, the portion of free PON1 in the plasma also acts as an antioxidant, but its hydrolysis capacity is reduced.

PON1 is anchored in the HDL3 fraction of high-density lipoproteins (HDL) in plasma [36]. The esterase activity of PON1 comprises the lactonase, homocysteine-thiolactonase (HTase) and arylesterase (AREase) activities [36]. The binding of PON1 to HDL in the bloodstream keeps all PON1 enzyme activities stable, Figure 3. Although most of the circulating PON1 is found in HDL, it can also be found in very-low density lipoprotein (VLDL) and postprandial chylomicrons [37]. PON1 can be transferred from HDL to VLDL and to circulating cells such as endothelial cells and macrophage that are in contact with HDL [31]. This enzyme maintains its end-N signal sequence, which is a hydrophobic part that binds the enzyme to HDL. The enzyme has two calcium-binding sites: One for enzyme stability and the other essential for enzymatic hydrolytic activity. Selective chemical modification of aspartic acid (D) and glutamic acid (E) residues with carbodiimides prevents Ca^2^+ binding and inactivates human PON1. It has three residual cysteines, in positions 353, 42 and 284. The first and second of these residues form a disulfide bridge though cysteine 284, participates in orientating PON1 or binds it to its substrate (6), and appears to be essential for the protective effect of PON1 against LDL oxidation [31,32,38].

PON1 has atheroprotective and anti-inflammatory properties [39]. PON1 inhibits the formation of oxidized LDL through hydrolysis of lactone ring in homocysteine thiolactone (HTL) molecule It can also degrade some oxidized lipids [39]. Indeed, PON1 modulates the metabolism of RNS, stimulates nitric oxide production and reduces macrophage foam cell formation [39].

The PON1 arylesterase and lactonase activities contribute to the maintenance the physiological functions of HDL in both cells and tissues. Changes in PON1 activities and HDL function have been associated with physiological conditions such as pregnancy and aging, as well as pathophysiological conditions such as atherosclerosis, diabetes, cerebrovascular and neurodegenerative diseases, iron overload, renal disease, drug metabolism and detoxification of organophosphate compounds [25,40,41,42,43]. Diet rich in fruits and vegetables, olive oils, polyphenols and flavonoids such as quercetin, increases the activity of the enzyme PON1, contributing to the reduction of oxidative stress in the degeneration process [44,45,46,47,48,49]. 

PON1 is a highly polymorphic gene with more than 400 single-nucleotide polymorphisms (SNPs) identified so far. However, only five common SNPs appear to have clinical relevance, three located in the promoter region (G-909C, A-164C and C-108T), and two in the translated region (L55M and Q192R). These polymorphisms have an impact on the activities and/or concentrations of PON1 [31]. PON1 concentration varies up to 13 times, while PON1 activity can vary by up to 40 times [37]. The SNPs of the PON1 promoter region seems to play the most important role in regulating the expression of the PON1 gene [31].

The PON1 Q192R polymorphism is located in the active functional site and can affect the enzyme stability and function [24]. This polymorphism is derived from the substitution of glutamine (G) by arginine (R) (GLn / Arg) at position 192 in the amino acid sequence [24]. The substitution results in increased hydrolysis of some organophosphate compounds such as paraoxon and chlorpyrifos-oxon (active metabolite of chlorpyrifos in vivo), but not toward phenylacetate [24]. In addition, it has been observed that individuals who have allozyme Q have greater protective efficiency against tissue damage caused by oxidized LDL (ox-LDL) compared to individuals with R allozyme [41,50]. On the other hand, the binding capacity between PON1 and HDL is lower in individuals with Q allozyme (approximately three times) when compared to R allozyme [41,50].

The SNP L55M PON1 occurs due to the substitution between nonpolar amino acids leucine (L) by methionine (M) at position 55 in the amino acid sequence of the protein [28]. Although it is still controversial whether SNP L55M can directly alter the hydrolase activity of PON1, it is known to change plasma concentrations of PON1 protein [28]. L55M has been shown to affect the structure of the PON1 protein [37]. Allozyme M is both more unstable and quickly proteolyzed when compared with L alloenzyme; these characteristics are differently associated with a decrease in enzyme concentration, activity and messenger RNA (mRNA) level of PON1 [37]. 

### 2.2. Paraoxonase 2

The PON2 isoform is highly expressed in several different types of human cells and tissues, mainly in macrophages and hepatocytes, lower lung airways, brain, cardiac and gastrointestinal systems. It is found in association with the endoplasmic reticulum and mitochondria, specifically associating with complex III of the inner mitochondrial membrane. PON2 deficiency alters mitochondrial function by decreasing mitochondrial complex I and III activity and total ATP levels and alters mitochondrial oxidative stress by increasing mitochondrial superoxide production, increasing lipid peroxidation and decreasing reduced glutathione levels. In vascular cells, PON2 has been found to be a cell-based enzyme and appeared in two glycosylated isoforms of approximately 40–43kDa. PON2 is not detectable in plasma [22,51,52].

Studies in animal models have shown that increased PON2 expression may protect against the formation of atherosclerotic plaque through modulation of intracellular oxidative stress. PON2 deficiency aggravates the development of atherosclerosis [53]. Moreover, cells transfected stably with overexpression of PON2 showed a decrease in intracellular oxidative stress caused by exposure to hydrogen peroxide. In brain tissue, PON2 is an antioxidant intracellular enzyme against oxidative stress. In CNS, PON2 expression has been found in nucleus accumbens, striatum and substantia nigra [54]. PON2 is found in astrocytes and neurons in different amounts. However, the loss of PON2 expression in both cells negatively modifies the cellular ability to recover from oxidative damage and subsequently death [55].

The SNPs G148A and C311S in PON2 gene, have been associated with the development of some diseases [56]. The PON2 C311S polymorphism does not change its expression and enzyme concentration in cells and tissues. On the other hand, the SNP PON2 C311S is associated with increased glycosylation of the enzyme and decreased lactonase activity [57]. PON2 C311S polymorphism has been associated with metabolic disorders of cholesterol transport, such as high plasma lipoprotein concentrations, coronary artery disease, complications in diabetes mellitus, ischemic stroke and Alzheimer’s dementia [57,58,59]. The SNP G148A PON2 has been associated with the development of cardiovascular disease, cerebrovascular disease and type two diabetes mellitus in different ethnic populations [60,61,62].

### 2.3. Paraoxonase 3

PON3 is an antioxidant hydrolase enzyme with approximately 40-kDa, synthesized in the liver. In plasma PON3 is bound to HDL and apolipoprotein-AI and possesses strong anti-oxidant properties but its concentration is about two orders of magnitude less abundant than PON1 [63]. PON3 is also expressed at low levels in the kidney [32]. PON3 was the last enzyme in the paraoxonase family genetic cluster to be described. Currently, very little is known about its function and physiological characteristics in humans. The enzymes PON3 and PON1 show some similarities in structure and hydrolase activity. Regarding the structure, both enzymes have three highly conserved cysteine (Cys) esidues in positions −41; −283 and −351 in the protein chain [64]. As for enzyme activity, PON3 can hydrolyze cyclic carbonate esters and lactones rapidly, mainly drugs such as statin lactones. The arylesterase activity of PON3 is almost undetectable when compared to PON1 [65].

PON3 participates in tissue homeostasis against oxidative stress in the same way as paraoxonases-1 and -2. Indeed, in vitro, PON3 hydrolyzes some products derived from oxidation process, such as both oxidized phospholipids and lipid (hydro)peroxides in ox-LDL, suppressing the oxidation propagation cascade in other lipids and phospholipids [66]. Indeed, previous studies have indicated that the decrease in the concentration of PON3 is associated with coronary artery disease, obesity and chronic liver disease [67,68,69]. In addition, in HDL particles from patients with systemic lupus erythromatosus and type one diabetes it was observed that the PON3 content was depleted, being associated with subclinical atherosclerosis [70]. Moreover, recent studies have described increased expression of PON3 in different types of tumor cells [56,71].

Currently there are six SNPs described in the promoter region of the PON3 gene: C-567T, A-665G, C-746T, G-4105A, T-4970G and A-4984G. These polymorphisms have little or no influence on the PON3 concentration [66].

## 3. Neurodegenerative Diseases

**Figure 4:**
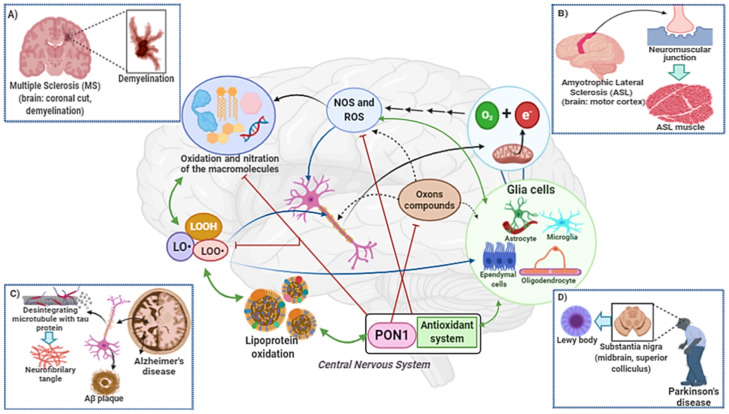
The consumption of oxygen by the brain is high. O_2_ is used in oxidative phosphorylation to produce energy in the form of ATP in mitochondria. ATP is used to maintain membrane potential, the flow of neurotransmitters and the synapse in neurons. During the ATP synthesis process, occurs electron (e-) leakage of the electron transport chain. The free electron reacts with oxygen and forms the superoxide radical (O_2_^•−^). Then, hydroxyl radical formation can occur from the Fenton and Haber–Weiss reactions. Reactive oxygen species (ROS) and reactive nitrogen species (NOS) react with macromolecules: Lipids, proteins, carbohydrates and DNA. Lipid peroxidation of polyunsaturated fatty acids results in the accumulation of lipid hydroperoxides (LOOH). In the degradation of LOOH, the formation of alkoxyl (LO•) and peroxyl (LOO•) radicals occurs, highly reactive with proteins and lipid members. This process is widespread and can occur anywhere in the brain. Initially, it changes neuronal and glial cell function, and then cell death. In addition, in the lipid peroxidation process cytotoxic aldehydes are formed. Cellular metabolism generates O2^•−^, H_2_O_2_ and NO•, that can react and oxidize neurotransmitters. Under physiological conditions, the CNS antioxidant systems (superoxide dismutase (SOD), catalase (CAT), glutathione (GSH) and PON1) are able to maintain tissue homeostasis. However, when dyshomeostasis occurs, either by an increase in toxic substances (such as organophosphate compounds) and/or oxidative stress, dysregulating lipid homeostasis, then the neurodegeneration process begins. Increased oxidative stress and decreased activity of antioxidant enzymes, such as PON1, are strongly associated with the pathophysiology of multiple sclerosis (**A**), amyotrophic lateral sclerosis (**B**), Alzheimer’s disease (**C**) and Parkinson’s disease (**D**).

The healthy human brain has about 100 billion neurons, which are interconnected by biochemical mechanisms called synapses. In this way, through the neuronal circuits of the brain, the cellular base of memories, thoughts, sensations, emotions, movements and skills are created. When irreversible changes occur in the brain niche, the neurodegeneration process begins, leading to the different types of neurodegenerative diseases, Figure 4. This process may be associated with changes in neurons, glial cells, as well as from metabolic changes, or systemic diseases that alter the permeability of the blood brain barrier (BBB) and can alter cognitive functions [72,73].

Thus, the brain environment becomes susceptible to pathological changes, with loss of cell function, cell death, increased neuroinflammation, oxidative stress and lipid peroxidation. Together, these factors affect both the biochemical and physiological properties of the myelin sheath [74]. The formation of myelin in the CNS is derived from the involvement of the macroglia plasma membrane around the axon. The brain structural composition consists of proteins (about 15–30%) and lipids (70–85%): Cholesterol (mostly non-esterified), phospholipids and glycolipids in a 2: 2: 1 ratio. In addition, the brain has about 20–30% of the body’s total cholesterol [74,75]. Cholesterol exchanges between the central nervous system and blood circulation is highly limited; this helps to avoid tissue damage and injury [75,76,77].

The association between cholesterol and neurodegenerative diseases is longstanding [78]. In fact, changes in lipid metabolism in the brain are associated with protein aggregation and the onset of senile plaque formation [79]. In addition, in several recent studies, cholesterol content and changes in the Apo-E gene have been associated with risk factors for worsening cognitive function and development of dementia [80,81]. Moreover, the Apo-Eε4 genotype has been associated with β-amyloid and tau protein aggregation, both associated with the development of dementia [82,83,84,85]. Interestingly, Thorvaldsson et al. [86] observed a non-linear association between total cholesterol concentration (low and high values) and worsening cognition. In addition, total cholesterol levels decrease over time and are associated with the rate of cognitive decline. On the other hand, Bennett et al. [87] did not find association between plasma total cholesterol and fractions, and plasma triglycerides with amyloid load in old age. However, it is possible that changes in lipid metabolism could occur in the CNS with no detected modifications in the blood circulation.

Apolipoproteins have been associated with neurodegenerative diseases, Table 1. The low activity of PON1 has also been associated with several neurodegenerative diseases as well as lipid changes and demyelination process (in an animal model) [38,40,88,89]. Here we review the studies, in humans, of the association between PON1 and four neurodegenerative diseases: Multiple sclerosis, amyotrophic lateral sclerosis, Alzheimer’s disease and Parkinson’s disease.

### 3.1. Multiple Sclerosis

Multiple sclerosis (MS) is characterized as a chronic, inflammatory and autoimmune neurodegenerative disease, causing demyelination of CNS axons and impairment of the ability to perform movement. The progression of the disease is slow and the survival of patients with MS is long. About two million people worldwide have MS [117]. MS is a heterogeneous disease that involves several immune-mediated metabolic pathways, associated with genetic, epigenetic and environmental factors. The cerebellum is the part of the brain where pathological changes occur due to MS. However, symptomatic changes are difficult to detect in the early stage of MS. Significant symptoms of MS start when the neurodegeneration process is progressing, associated with excessive inflammation in the CNS [118,119,120,121]. The neurodegeneration process is characterized by the pathological presence of focal areas of demyelination or plaques. In the affected region, the BBB breaks down, allowing the infiltration of cells, mainly B lymphocytes, cells of the monocyte-phagocytic system, dendritic cells. Then, these cells activate signaling cascades that induce differentiation of memory T lymphocytes into pro-inflammatory auxiliary T lymphocytes, mainly cells T helper 1 and T helper 17. This is followed by an intense inflammatory process with an increase of effector cells in the CNS parenchyma associated with an increase in inflammatory mediators, leukocyte recruitment and increased expression of endothelial adhesion molecules. Proinflammatory stimulation of microglia cells leads to destruction of the myelin sheath in neurons present in the white and gray substance associated with changes of CNS lipid metabolism [120,122,123,124,125,126,127]. 

The association between oxidative damage and PON1 activity was first assessed in MS by Ferretti et al. [128]. The authors observed an association between increased levels of cholesterol ester hydroperoxides (CE-OOH), a product of the oxidation of lipoprotein-containing cholesterol, and decreased plasma paraoxonase activity in 24 individuals with MS. Indeed, Jamroz–Wisniewska et al. [129] have observed that PON1 activity was significantly lower in patients with recurrent-remitting multiple sclerosis (RRMS) in relapse compared to other types of MS (RRMS patients in remission and progressive patients with RRMS), and that hypercholesterolemia was observed in patients with MS. Moghtaderi et al. [130] have shown that the serum paraoxonase and arylesterase activities of PON1 were not different between patients with relapsed RRMS and healthy individuals, and that there was no difference in the distribution of SNP Q192R. Oxidative stress contributes to the pathogenesis of multiple sclerosis and are associated with increased demyelination of motor neurons [131,132,133]. In patients with RRMS it has been observed that serum activities of PON1 and total antioxidant status were low, while the total oxidant status was high [134]. Palavra et al. [135] evaluated the presence of cardiovascular risk factors in 30 RRMS individuals with the clinical activity of the disease (assessed by the expanded disability status scale (EDSS)). Individuals with RRMS showed increased serum triglycerides, ox-LDL and ox-LDL/LDL ratio, and reduced HDL levels. The concentration of ox-LDL was correlated with EDSS and vascular endothelial growth factor (VEGF). PON1 activity did not change and was not associated with any lipid marker [135]. 

Homocysteine hiolactone (HTL) is a cyclic homocysteine thioester (Hcy). HTL reacts spontaneously with lysine protein residues, modifying the physiological and structural functions of proteins. PON1 can hydrolyze the lactone ring in thiolactone homocysteine to thioester homocysteine [8,36]. Jamroz–Wiśniewska et al. [136] evaluated the level of plasma Hcy, HTL and autoantibodies against N-homocysteine proteins, and PON1 activity in 61 patients with MS, in outbreak-remission (*n* = 25) and in the secondary-progressive type (*n* = 36). The results were compared to a healthy group composed of 44 individuals. The homocysteine level was higher in both groups with MS. Concentration of HTL tended to be increased in patients with outbreak-remission. However, the anti-N-homocysteine antibodies titer was similar among groups. PON1 activity was lower in patients with secondary-progressive multiple sclerosis compared to the other groups. Cladribine is an analogue derived from purine, used to treat the recurrent form of multiple sclerosis. Treatment with cladribine decreases the number of B and T lymphocytes, especially T CD4 + and T CD8 +, in order to decrease inflammation and infiltration of lymphoid cells in the CNS [137]. Jamroz–Wiśniewska et al. [138] showed that treatment with subcutaneous cladribine did not change any of the activities of PON1 (paraoxonase, arylesterase and lactonase), even with a decrease in Hcy levels [139]. 

In a small study with 27 individuals with multiple sclerosis that received an isocaloric and ketogenic Mediterranean diet for 4 months it was observed an increase in PON1 activity [140]. In addition, in an observational study (*n* = 57) it was demonstrated that nutrient intake by patients with MS has a disequilibrium of the macronutrients that favors abdominal obesity associated with increased concentration of pro-inflammatory interleukin-6 [141] PON1 activity was reduced, but it was not correlated with high pro-inflammatory values [141].

Sidoti et al. [142] investigated the association between polymorphisms of the enzymes PON1 (Q192R and L55M) and glyoxalase I (GI) (GI A111E) with the susceptibility to MS in 209 individuals with RRMS paired with 213 peoples clinically healthy. The frequency of the LM heterozygous genotype (SNP L55M PON1) was high (approximately 50%) in individuals with RRMS [142]. Moreover, the presence of the homozygous MM genotype (SNP L55M PON1) resulted in an elevation of MS risk of about 2.8 times. The association between L55M and A11E polymorphisms has shown that Glo1EE/PON1 55MM and Glo1AE/PON1 55LM can contribute to an intermediate and high risk susceptibility to MS in an Italian population [142]. 

However, a study carried out in a Spanish Caucasian population composed by 228 individuals with multiple sclerosis and 220 healthy controls found no differences in the frequency distribution of the PON1 genotypes (Q192R and L55M) [143]. The PON1-55L and PON1-192R alleles were not associated with the risk of developing MS [143]. In addition, no difference have been observed in the frequency L55M, Q192R and A-162G in PON1 gene and C311S PON2 gene polymorphisms in Polish population with multiple sclerosis [144]. 

### 3.2. Amyotrophic Lateral Sclerosis 

Amyotrophic lateral sclerosis (ALS) is a neurodegenerative disease with one of the worst known medical prognosis due to degeneration, loss of function and death of motor neurons present in the cerebral cortex, brain stem and spinal cord, that control the movements of voluntary muscles. The progressive loss of neurons results in debilitating motor weakness, and increased paralysis. [145,146,147]. The survival time is very short, around 2–5 years after the onset of motor symptoms. Currently, ALS is classified into two forms due to its etiology: Familial and sporadic. Familial form is characterized by an autosomal dominant mutation in the gene encoding the antioxidant enzyme SOD1; this alteration is present in approximately 20% of the cases diagnosed with ALS in the world. In these patients there are considerable increase in oxidative stress. However, the cause of the development of sporadic ALS remains unknown [145,146,147]. Although some studies indicate as risk factors for sporadic ALS the exposure to pesticides in crops and in the rural environment; occupational exposure to heavy metals, such as cadmium and toxic products, smoking and others, there is still no strong evidence to establish which risk factor is decisive for the development of ASL [22,147,148,149,150,151]. It is possible that the association of a set of factors, mutations, epigenetic changes, polymorphisms can lead to the deterioration of motor neurons and astrocyte dysfunction. Indeed, oxidative stress in the CNS plays a central role in the pathophysiology of ALS [22,147,148,149,150]. 

The association of PON1 polymorphisms and the development of ALS began after the observation of the high incidence of sporadic ALS in young veterans of the Gulf War [152]. In fact, during the Gulf war, veterans were exposed to organophosphate pesticides, nervous agents, DEET (N, N’-Diethyl-3-methylbenzamide) insect repellent and use of pyridostigmine. All of these factors contribute to the increase in chronic diseases [153]. In addition, the frequency of the R allele (QR or RR) of Q192R PON1 polymorphism was found to be higher in veterans who had clinical symptoms associated to psychoneurological dysfunction. Therefore, the low arylesterase activity of PON1 type Q in veterans was correlated with advanced acute toxicity after taking pyridostigmine [154]. Type Q is the POase-AREase allozyme that easily hydrolyzes oxon compounds [154]. In addition, the Q192R PON1 polymorphism was studied in 116 patients with gender-matched sporadic ALS with 437 healthy characteristics. A higher frequency of the R allele was observed in patients with sporadic ALS; the risk to develop sporadic ALS was 1.8 times higher in individuals with R allele. However, no association was observed between SNP Q192R and others clinical variables of ALS [155]. 

ALS is a multifactorial disease characterized by cerebral cell dysfunction and mitochondrial alteration. It is associated with the progressive increase in neuroinflammation, generalized oxidative stress and metabolic alterations [156,157]. Saeed et al. [158] have shown a significant link between the polymorphisms present in the PON gene and ALS. The authors have identified a high linkage disequilibrium in PON2 and PON3 genes, and presence the polymorphism rs10487132 (INS2 + 3651A > G) in PON3 and rs11981433 (T > C/G) in PON2. The G allele of PON3 (INS2 + 3651A > G) was associated with sporadic ALS among descendants of the same family [158]. The C allele of the C311S PON2 and R allele of the Q192R PON1 polymorphism were associated with sporadic ALS. The presence of the R-C haplotype has been linked to the development of ALS [155]. In addition, the expression of messenger RNA of the PON2 gene was decreased in spinal cord and trunk tissue of patients with ALS, and PON1 was undetectable [159].

Morahan et at. [160] investigated the gene-environment interaction between seven PON1 polymorphisms in 143 patients with sporadic ALS paired with 143 controls. In this study, the PON1 L55M, Q192R and I102V coding polymorphisms were evaluated, as well as the polymorphisms of the promoter region that can alter the expression of PON1: C-909G, G-832A, G-162A and C-108T. Interestingly, authors analysis showed that exposure to pesticides provided a risk for developing ALS. However, when exposure was present in addition to the susceptibility allele, the chance of having the disease increased by approximately two times for SNPs G-832A, G-162A and C-108T, and in low doses of pesticides the SNP: G-162A. On the other hand, Q192R presented a higher risk of ALS only in individuals exposed to high doses of herbicide or pesticide associated with the R allele. It is important to note that this study found an association between allele and susceptibility to ALS. There was no interaction between genotype and/or haplotype with sporadic ALS. In this study, L55M SNP was not associated with the chance of developing ALS [160].

Controversially, in an Irish population, an association was found between SNP L55M PON1 (55M) and PON3 (INS2 + 3651 G), with sporadic ALS [161]. Moreover, Diekstra et al., [162] evaluated the association between rural and urban environment with SNP L55M PON1 in 98 individuals with ALS [162]. They have observed an association between the exposure environment and the SNP L55M. Individuals with ALS residents in the rural area showed a higher frequency of the M allele, and a lower survival when compared to residents of the urban area [162].

In addition, Wills et al. [163] observed a high frequency of R allele (Q192R) in individuals with ALS. However, there was no difference in PON1 protein levels, or in paraoxonase, arylesterase or diazoxonase activities, and the rates of organophosphate hydrolysis had no effect on the survival of patients with ALS. Landers et al. [164] described the polymorphism rs987539 (C > T) in PON2 gene and rs2074351 (G > A) in PON1 gene associated to the sporadic ALS. Furthermore, the authors identified that the presence of haplotype sequence GACGT in the polymorphisms (rs854565 (A > C/G/T), rs2299261 (A > G), rs705381 (T > C), rs705382 (C > A/G) and rs4141217 (C > T)) are a risk for the development of sporadic ALS [164]. 

Valdmanis et al. [165] conducted a study in three different countries (France, Canada and Sweden) to check the association of the PON gene cluster with ALS. A SNP haplotype was found in the C-terminal portion of PON2 that included the alteration of amino acids PON2 C311S in the French and Canadian population, as well as in the other combined populations. The casuistic stratification have shown that this alteration was a relevant risk factor for the development of ALS, regardless of the patient nationality [165]. In addition, seven mutations found in the PON gene in patients with familial and sporadic ALS were observed [166]. 

However, in an Italian population, the SNPs L55M, Q192R in PON1 and C311S in PON2, both genotype and haplotype, were not associated with ALS [167]. Furthermore, in a study with nine polymorphism present in the PON gene cluster, Q192R; L55M; C-162T; rs705382(C > A/G); rs854548(A > C/G/T) and rs757158 (C > T) in PON1 gene, rs7493 (G > C) and rs11981433 (T > C,G) in PON2 gene and INS2+3651A > G in PON3 gene, in the Chinese population, no association was found between these SNPs and sporadic ALS [168]. Similar results were observed in a Dutch population [169]. However, in two meta-analyses [170,171], one of them stratified for the European population [171], did not confirm the association between SNPs (PON1 Q192R), (PON1 L55M), (INS2+3651A > G (PON3)) and ALS. These results reinforce the need for larger and more robust research, as well as the association of genetic determinants and their interaction with the environment in susceptibility to ALS [172,173]. 

Recently, Verde et al. [174] described that the SNP Q192R PON1 can act as a modifier of the sporadic ALS phenotype. Individuals that had the homozygous genotypes RR and heterozygous GQ had a lower survival rate when compared to the homozygote genotype QQ. Moreover, the allele R was associated with bulbar onset.

### 3.3. Alzheimer’s Disease

Dementia is characterized by a progressive decline in cognitive functions. Among the dementias diagnosed in the elderly, about 80% correspond to Alzheimer’s disease (AD). Individuals diagnosed with AD present changes in the hippocampus and cortical structures, with dysregulation of the cholinergic system in the CNS [175,176,177]. Currently, there are two forms of AD: The family form with early onset (<65 years) and the form of late onset, or sporadic. The definitive diagnosis of AD is performed in post-mortem histological examinations [178,179]. In histopathological analysis, the presence of amyloid plaques in the extracellular region and/or throughout the brain tissue is observed due to deposition and aggregation of the β-myeloid peptide (Aβ) (Aβ1–40 and Aβ1–42) [180,181]. In the intracellular medium of neurons, deposition of neurofibrillary tangle formation (NFT) occurs due to incorrect folding and hyperphosphorylation of the Tau protein [180,181,182]. The increase in senile plaques interferes with the synaptic signal and neuron–neuron communication. Tangles of tau block the transport of nutrients, as well as alter intracellular signaling. Both processes lead to loss of cell function, followed by neuronal death [180,181,182,183]. About 1% of the diagnosed cases of AD are associated with genetic mutations. Therefore, the most frequent mutations are associated with the amyloid precursor protein (APP) gene and the genes for the proteins presenilin 1 and presenilin 2. Individuals with mutations in any of these three genes are likely to develop Alzheimer’s symptoms before 65 years old. In addition, the Apo-E gene isoforms (ɛ2, ɛ3 and ɛ4) are associated with disease, mainly with isoforms ɛ4 [184,185,186,187]. 

Cardiovascular diseases and lipid metabolism alterations are predictors for the development of Alzheimer’s disease. The association between the PON1 enzyme and the development of AD was described in the late 1990s [188,189]. In addition, treatment with statins have shown to improve the cognitive function in patients with AD [190,191,192,193]. Paraoxonases-1 and -2 are expressed in the human frontal cortex [194]. PON2 mRNA levels are regulated positively in AD compared to controls without dementia [194]. In addition, a synergism has been observed between SNPs PON1 Q192R and L55M and apolipoprotein-E (Apo-E) for susceptibility to AD, as well as vascular dementia (VD) [195]. An interactive association was described between the presence of the allele S (SNP C311S PON2) and ε4 allele of apolipoprotein-E in AD and VD [196]. However, there is a divergence between studies associating this SNP with dementia [197]. In the Chinese population with AD, the frequency of the PON2 C allele was higher, and there was no association with the Apo-Eε4 allele of apolipoprotein-E [198]. 

The cluster of PON genes is highly polymorphic, being able to present wide variability and different frequencies between ethnicities, as well as in different diseases. The Q192R polymorphism was a discriminating factor between AD and VD; however, there was no difference in genotypic distributions between groups [199]. On the other hand, the association between Q192R, Alzheimer’s disease and coronary artery disease (CAD) is controversial. Scacchi et al. [200] have observed a low frequency of the R allele in individuals with AD; the adjustment for age, gender and polymorphism of Apo-Eε4 highlighted that the genotype PON1 RR was a protective factor for AD, whereas for young individuals with CAD this genotype was associated with a risk factor [200]. Similar results for the R allele have been reported in the Chinese population. The presence of the R allele indicated a protective factor against the development of AD [201]. However, in older Singaporean Chinese patients, the R allele was associated with a worse functional state, the presence of neuropsychiatric symptoms and severe advanced dementia in patients with mixed dementia [202]. Controversially, in a French population, the R allele seemed to be a risk for dementia, together with the T allele (C-107T), independently of the Apo-Eε4 allele [203]. However, SNP Q192R of the PON1 gene has not been associated with risk of AD in Italian and Polish populations [204,205]. 

PON1 is an exogenous acetylcholinesterase inhibitor (ChEI) [206]. The influence of SNP Q192R in response to treatment with ChEIs has been evaluated in a small cohort of patients with AD [207]. Apparently, individuals with AD and the R allele had a better response to therapy compared to homozygous QQ individuals [207]. The authors pointed out that the allele was associated with a greater capacity for hydrolysis of the enzyme; for this reason, there may be a synergism in the metabolism of drugs such as donepezil, galantamine and rivastigmine, which may improve their effectiveness [207]. On the other hand, another study with three PON1 SNPs (Q192R, L55M and A−162G) showed no change in response to treatment with acetylcholinesterase inhibitors in patients with AD [208]. Several factors are able to change the therapeutic response to acetylcholinesterase inhibitors. However, association studies of PON1 enzymatic activity and polymorphisms with environmental exposure, eating habits, genetic factors of susceptibility to AD, drug metabolism-associated polymorphism, are lacking [209,210,211]. 

In patients with Alzheimer’s disease, the homozygous TT genotype (PON1 C-107T) was associated with a change in the distribution of lipoprotein cholesterol with a higher prevalence of a smaller and denser LDL [212]. It has also been associated with increase of plasma oxidized LDL levels [212]. Oxidative stress in Alzheimer’s disease contributes to lipoprotein oxidation, increased neuroinflammation, neuronal loss and endothelial damage [213,214]. However, these are multifactorial mechanisms that cannot be attributed to the presence of a single polymorphism. In addition, in an Italian case-control study, no association was observed between the T allele (C-107T) and the development of AD. Indeed, no Apo-Eε4 genotype association was found [215]. Another polymorphism in promoter region gene PON-1, the SNP C-108T, has been associated with the development of AD. The T allele was more frequent in patients with AD, and the homozygous genotype (TT) was associated with low arylesterase activity of PON1 [216,217]. However, the relationship between AD and the SNP PON1 C-107T and C-108T still needs to be clarified. 

Erlich et al. [58] genotyped 29 SNPs in the PON gene region in a large cohort composed of Afro-descendants and Caucasians with AD. It was observed that the location of positive associations for the development of AD were found in distinct regions in the PON gene in both ethnicities. Sliding window haplotype analyses showed that SNP C-161T was associated with AD; however, SNP C-161T was not associated with AD in the French AD population [218]. In addition, the authors established an association pattern in which the presence of the T allele had a deleterious effect, independently or in association with other genotypes [58]. Moreover, in this study it was shown that these SNPs, A-107G, Q192R, L55M in PON1 and C311S in PON2, before associated with AD risk may not act independently, but rather in linkage disequilibrium with other polymorphisms that are associated with the pathophysiology of AD. These results can partially explain the inconsistencies among the studies that investigate PON1 Q192R and L55M polymorphisms as associated with the development of Alzheimer’s disease. 

In the brain tissue of AD patients, a high frequency of the homozygous genotype MM PON1 L55M was observed [219]. In addition, patients with homozygous MM had a 2.5-fold increase in the proportion of Aβ42/Aβ40 in the frontal cortex compared to control and individuals with AD carrying LL genotype [219]. Moreover, AD patients with R allele (Q192R) had a significantly lower Aβ42 / Aβ 40 ratio compared to Q192Q homozygous AD patients [219]. In addition, individuals with M allele (L55M PON1) showed a decrease in the total amount of nicotinic receptor and choline acetyltransferase (CHAT) activity in the temporal cortex [219]. A large cohort of clinical cases confirmed this study by AD autopsy (*n* = 1.066) [220]. The M allele of the L55M SNP was associated with a risk of developing AD in men. Men and women with the MM-QQ genotype have higher survival rate (about 2.5 years) and later age of disease onset (about 1.5 years). In addition, AD individuals with R allele had a decrease in both, Aβ42 levels and Aβ42/Aβ40 ratios. In the hippocampus and frontal cortex, patients with MM genotype showed a decrease in their Aβ40 concentration and an increase in Aβ42/Aβ40 ratios compared to both, LM and LL genotypes. In men with MM genotype it has been observed more neuritic senile plaques than those with LL genotype in the fusiform gyrus and frontal cortex [220]. On the other hand, in the meta-analysis, individuals with the PON1 polymorphisms Q192R and L55M were not susceptible to AD [221].

In fact, PON1 activity is low in different forms of the dementia [40,199,222,223,224,225,226]. The reduced activity of PON1 was associated with an increase in the atherosclerotic process in patients with AD [227]. A decrease in paraoxonase activity in patients with AD was associated with Apo-Eε4 isoforms and both total cholesterol and elevated LDL-cholesterol [228]. Indeed, the ratio between PON1 and platelet activating factor acetyl hydrolase (PAF-AH) activity was correlated to the increase in oxidized LDL [229]. Moreover, 8-hydroxy-2’-deoxyguanosine (8-OHdG), an oxidized product derived from deoxyguanosine, formed after the oxidation process in DNA, has been negatively correlated with PON1 activity in AD patients [230]. However, the proportion between arylesterase PON1 activity and Apo-AI showed an inverse relationship with the concentration of both total and phosphorylated tau proteins in patients with AD in cerebrospinal fluid [231].

### 3.4. Parkinson’s Disease

Parkinson’s disease (PD) is featured by a strong decrease in the production of dopamine in substantia nigra, due to degeneration of dopaminergic neurons. This process is slow; initially there is an impairment of the motor system, and in more advanced cases non-motor symptoms are observed. Currently, about 1% of people in the world aged over 60 years develop PD. Clinically, patients show changes in the motor system, such as bradykinesia, rest tremor and stiffness, symptoms known as parkinsonism [232]. However, critically ill patients have non-motor changes, including anosmia, constipation, pain, anxiety, depression and psychosis. Initially, cognitive disorders are mild, evolve to moderate, and then progress to dementia [233]. The pathophysiological characteristics of PD include a slow and progressive degeneration of dopaminergic neurons, depletion of striatal dopamine, disappearance of neuromelanin and the appearance of intracellular Lewy bodies, derived from the incorrect folding of α-synuclein protein [234,235]. During the progression of PD there is an increase in lipid (hydro) peroxidation and altered mitochondrial function, due to electron leakage, and consequent formation of the hydroxyl radical and hydrogen peroxide, associated with the exhaustion of the redox system. These factors together contribute to increased dopamine oxidation in the synaptic cleft and to the neuronal death leads development of dementia [236,237,238].

The association between Parkinson’s disease and the PON1 enzyme is due to the fact that toxic metabolites such as dopaminergic neurotoxin, 1-methyl-4-phenyl-1,2,3,6-tetrahydropyridine (MPTP), have been associated with development of PD. MPTP has a chemical structure similar to some organophosphates [239,240]. Moreover, organophosphates are bioactivated in cholinesterase inhibitors after metabolization by cytochrome P 450 systems, and the oxon (toxic) form is hydrolyzed by PON1. In addition, the B allele of the SNP Q192R PON1 has been associated with the development of PD in a Japanese population [241]. However, the association between SNP Q192R was not associated with the development of PD in other populations, such as Caucasian and Chinese population [242,243,244]. 

SNP L55M was considered an independent risk factor for the development of PD in different populations. The frequency of the M allele was higher in Parkinson’s patients, and the estimated relative risk was approximately two times higher when compared with homozygous individuals for the L allele [245,246]. Additionally, environmental exposure to diazinon, chlorpyrifos and parathion in individuals with homozygous genotypes QQ and MM (SNPs Q192 and L55M, respectively), have been associated with Parkinson’s development by up to three times [247]. Indeed, the frequent use of organophosphate chemicals was associated with PD at a chance of up to 71%. Individuals with both homozygous genotypes MM and QQ, had an approximately six-fold chance of developing PD [248]. The presence of these polymorphisms characterizes a “slow metabolization” of organophosphates. However, in other studies, the association between PON1 and PD polymorphism has not been observed [205,249,250,251].

Polymorphism in the PON1 G-832A promoter region was associated with PD. The A allele was more common among controls than in PD patients, and may have a protective effect [252]. In addition, SNP G-832A was in an imbalance with PON1 C-909G polymorphism. [252]. The polymorphism present in the PON1 promoting region, C-909G, has been associated with increased expression of the PON1 gene [253].

In patients with PD living in a rural area exposed to pesticides, the serum activities of acetylcholinesterase (AChE) and PON1 were reduced. A linkage disequilibrium was observed between the PON1 and the AChE locus. The polymorphism of the PON1 C-108T promoter region and the AChE deletion (ΔAChE) were associated with Parkinson’s development by approximately two times [254]. The authors suggested that hereditary interaction at the AChE and PON1 locus may increase the occurrence of insecticide-induced Parkinson’s disease [254]. Serum reduction in total cholesterol, LDL, PON1 and urate were associated with PD progression. In addition, serum ferritin concentration was inversely correlated with PON1 activity [255]. The association between ferritin and PON1 may be a link between inflammation and the enzymatic antioxidant system [43]. Decreased serum paraoxonase activity in PD patients has been associated with increased oxidative stress, lipid peroxidation and changes in iron metabolism markers [256,257,258]. 

## 4. Conclusions

PON1 activity and polymorphisms have been associated with neurodegenerative diseases. However, taken into consideration that PON1 may have plasma activities that do not reflect the enzyme activity in the central nervous system, relatively little is known to date about the real role of PONs in the central nervous system or their mechanism of action. Data have suggested that the Q192R and L55M intronic polymorphisms are risk factors for the development of neurodegenerative diseases. However, several other studies describe contradictory results. 

Although the role of PONs is described as hydrolases enzymes in several diseases, robust studies are still lacking to clarify the association between polymorphisms of the PONs gene cluster, including PON2 and PON3, and enzymatic activities in the neurodegeneration process. Studies at the cellular level are necessary to understand the physiological functions of PONs in macro and microglia cells and in neurons. Differences in the distribution and specificities of PONs in the human body indicate that there may be specific tissue activity for each enzyme. 

Nevertheless, taken all studies together, paraoxonase seems to have a role in the decrease and/or prevention of the neurodegeneration process associated with the imbalance of the redox system. Further studies in this direction could provide enough information that would lead to new clinical-pharmacological interventions.

## Figures and Tables

**Table 1:** Apolipoproteins and neurodegenerative diseases.

Apolipoprotein	Synthesis	Lipoprotein	Function	Disease	Reference
**Apo-AI**	Intestine and liver	HDL	Cofactor for LCAT. Binding to the ABC-A1.	Infantile neuronal ceroid lipofuscinosis (INCL) (animal model); Alzheimer’s disease; Amyotrophic lateral sclerosis	[90,91,92]
**Apo-AII**	Liver	HDL	Atheroprotection	Senile amyloidosis (mice); Senile dementia;	[93,94]
**Apo-AIV**	Liver	CM; VLDL; HDL	Reverse cholesterol transport	Huntington’s disease	[95]
**Apo-B48/B100**	Small intestine; Liver	CM; VLDL; IDL; LDL	Formation of chylomicrons. Ligand for LDL receptor	Early-onset Alzheimer’s disease (EOAD); Syphilitic dementia; Cerebral Amyloidosis; Amyotrophic lateral sclerosis	[90,96,97,98]
**Apo-CI**	Liver	VLDL; HDL	Minor LCAT activator.	Behavioral variant Frontotemporal dementia (bvFTD); Alzheimer’s disease	[88]
**Apo-CII**	Liver	CM; VLDL; HDL	Activator of lipoprotein lipase.		
**Apo-CIII**	Liver	CM; VLDL; HDL	Inhibition of the clearance of TG-rich lipoproteins and lipoprotein lipase.	Mild cognitive impairment	[99,100]
**Apo-D**	CNS: Neurons, oligodendrocytes, and astrocytes.	HDL	Participation in lipid transport during both degeneration and regeneration processes.	Alzheimer’s disease; Multiple sclerosis; Parkinson’s disease;	[101,102,103]
**Apo-E**	Liver	CM; VLDL; HDL	Removal of Apos from plasma.	Alzheimer’s disease; Amyotrophic lateral sclerosis; Multiple sclerosis	[80,104,105,106,107,108,109,110,111]
**Apo-J/Clusterin**	CNS:(ependymal cells and some neurons)	HDL	Protection against oxidative stress.	Parkinson’s disease; Alzheimer’s disease; Multiple sclerosis	[112,113,114,115,116]

Abbreviation: ABC: ATP-binding cassette; CNS: Central nervous system; CM: Chylomicron; HDL: High-density lipoprotein; LDL: Low-density lipoprotein; IDL: Intermediate-density lipoprotein; VLDL: Very low-density lipoprotein and LCAT: Lecithin–cholesterol acyltransferase.
